# AMG853, A Bispecific Prostaglandin D_2_ Receptor 1 and 2 Antagonist, Dampens Basophil Activation and Related Lupus-Like Nephritis Activity in Lyn-Deficient Mice

**DOI:** 10.3389/fimmu.2022.824686

**Published:** 2022-04-04

**Authors:** Christophe Pellefigues, John Tchen, Chaimae Saji, Yasmine Lamri, Nicolas Charles

**Affiliations:** ^1^ Université de Paris, Centre de Recherche sur l’Inflammation, INSERM UMR1149, CNRS ERL8252, Faculté de Médecine site Bichat, Paris, France; ^2^ Université de Paris, Laboratoire d’Excellence INFLAMEX, Paris, France

**Keywords:** SLE, AMG853, PGD_2_, basophils, PTGDR

## Abstract

Systemic lupus erythematosus is a complex autoimmune disease during which patients develop autoantibodies raised against nuclear antigens. During the course of the disease, by accumulating in secondary lymphoid organs (SLOs), basophils support autoreactive plasma cells to amplify autoantibody production. We have recently shown that murine lupus-like disease could be controlled by 10 days of oral treatment with a combination of prostaglandin D_2_ (PGD_2_) receptor (PTGDR) antagonists through the inhibition of basophil activation and recruitment to SLOs. Importantly, inhibiting solely PTGDR-1 or PTGDR-2 was ineffective, and the development of lupus-like disease could only be dampened by using antagonists for both PTGDR-1 and PTGDR-2. Here, we aimed at establishing a proof of concept that a clinically relevant bispecific antagonist of PTGDR-1 and PTGDR-2 could be efficient to treat murine lupus-like nephritis. Diseased Lyn-deficient female mice received treatment with AMG853 (vidupiprant, a bispecific PTGDR-1/PTGDR-2 antagonist) for 10 days. This led to the dampening of basophil activation and recruitment in SLOs and was associated with a decrease in plasmablast expansion and immunoglobulin E (IgE) production. Ten days of treatment with AMG853 was consequently sufficient in reducing the dsDNA-specific IgG titers, circulating immune complex glomerular deposition, and renal inflammation, which are hallmarks of lupus-like disease. Thus, bispecific PTGDR-1 and PTGDR-2 antagonists, such as AMG853, are a promising class of drugs for the treatment or prevention of organ damage in systemic lupus erythematosus.

## Introduction

Systemic lupus erythematosus (SLE) is a chronic remitting–relapsing autoimmune disease affecting mainly women of child-bearing age (1). These relapses are associated with the increased detection of autoreactive immunoglobulin titers [of immunoglobulin G (IgG), IgA, IgM, and IgE isotypes] mainly raised against nuclear antigens such as double-stranded DNA (dsDNA) or ribonucleoproteins (RNPs) (2). Autoreactive antibodies can form circulating immune complexes (CICs) together with autoantigens and complement factors, the deposition of which in target organs can lead to chronic inflammation and organ failure (3). About 25%–50% of SLE patients develop lupus nephritis, which is evidenced by the glomerular deposition of CIC and which can evolve toward fibrosis, glomerular dysfunction, and kidney failure (4). No efficient specific treatment is currently available for SLE patients, and flares of the disease are usually contained with high doses of corticosteroids and immunosuppressive drugs that are not devoid of serious side effects. Maintenance therapy is recommended after lupus nephritis flares to prevent further relapses and end-stage renal disease. These immunosuppressive therapies can be deleterious and are associated with high morbidity (4, 5). There is an urgent need to develop safe alternatives to maintain the remission state and/or prevent the occurrence of kidney involvement in SLE patients.

Autoantibodies and CICs are considered the main pathogenic factors in the pathophysiology of SLE. Beyond their direct effects on the targeted organs, CICs can activate some innate immune cells through Fc receptors and/or nucleic acid receptors. For instance, CICs induce the production of type I interferon (IFN) by plasmacytoid dendritic cells, the production of B-cell-activating factor of the tumor necrosis factor (TNF) superfamily (BAFF) by monocytes and macrophages, and the release of autoantigens by neutrophils through NETosis (neutrophil extracellular traps). Thus, these immune cells participate in the amplification of autoantibody production by providing key cytokines or antigens to autoreactive B and T cells (1, 3).

We previously showed that basophils contributed to SLE disease amplification by promoting the production of autoantibodies after their accumulation in secondary lymphoid organs (SLOs), both in several lupus-like mouse models and in SLE patient cohorts (6–10). In addition, we demonstrated that IgE, autoreactive IgE, and type 2 immunity contributed to the pathophysiology of lupus disease both in lupus-like mouse models and in SLE patients (8, 10–13). Basophils can be activated by numerous inflammatory mediators, including prostaglandin D_2_ (PGD_2_), the titers of which were increased in active SLE patients and lupus-prone mice (10). Indeed, combined treatment with antagonists targeting each PGD2 receptors (PTGDR), e.g., PTGDR-1 (laropiprant) and PTGDR-2 (CAY10471), was sufficient in reducing basophil recruitment to SLOs, plasmablast accumulation, autoreactive antibody production, CIC glomerular deposition, and kidney inflammation in less than 10 days, in both genetic spontaneous and inducible lupus-like nephritis mouse models (10). However, these effects could have been due to particular features of the antagonists used. Indeed, laropiprant showed inverse agonist and pharmacochaperone properties by inhibiting PTGDR-1 constitutive cAMP production and by stabilizing its expression in the plasma membrane (14), while CAY10471 showed an extremely low dissociation rate from PTGDR-2 (“insurmountable” antagonist) (15). AMG853 (vidupiprant) is a bispecific antagonist of both PTGDR-1 and PTGDR-2 without such particular properties that showed good safety and tolerability profiles in clinical trials (16). AMG853 could represent a promising alternative to preventing or limiting basophil accumulation in SLOs and breaking the basophil-dependent amplification loop of autoantibody production.

Here, we evaluated the efficacy of AMG853 in dampening the lupus-like disease in aged *Lyn* ^−/−^ female mice. AMG853 treatment reduced basophil and plasmablast accumulation in SLOs, autoreactive antibody titers, CIC glomerular deposition, and the kidney inflammation in this model. Overall, we established a proof of concept that AMG853, a clinically relevant bispecific PTGDR-1 and PTGDR-2 antagonist, can control lupus-like inflammation in a manner similar to the combination of PTGDR-1- and PTGDR-2-specific antagonists.

## Materials and Methods

### Mice and Treatments

C57BL/6J wild-type (WT) mice were purchased from Charles River Laboratories. *Lyn* ^−/−^ mice (17) on a pure C57BL/6J genetic background were bred in our local animal facility in specific and opportunistic pathogen-free conditions and maintained in specific pathogen-free conditions during the experiments. For *ex vivo* experiments, spleen from 10- to 12-week-old WT mice were used. Only 40- to 50-week-old female mice were used in the *in vivo* experiments. Mice received treatment by oral gavage with 14 mg kg^−1^ day^−1^ of AMG853 (Tocris, Bio-Techne, Noyal Châtillon sur Seiche, France) or vehicle (10% ethanol in tap water) daily for 10 days. Blood was harvested under isoflurane anesthesia in the retro-orbital sinus on day −1 of the treatment procedure or by intracardiac puncture immediately after sacrifice on day 10. Mice were euthanized in a controlled-released CO_2_ chamber. The study was conducted in accordance with the French and European guidelines and was approved by the local ethics committee comité d’éthique Paris Nord no. 121 and the Ministère de l’enseignement supérieur, de la recherche et de l’innovation under the authorization number APAFIS#14115.

### Human Sample Handling

Blood samples were collected from adult healthy volunteers. The study was approved by the Comité Régional de Protection des Personnes (CRPP, Paris, France) under the reference ID-RCB 2014-A00809-38. Written informed consent was obtained from all individuals. All samples were collected in heparin blood collection tubes (Becton Dickinson, Franklin Lakes, NJ, USA) and processed within 2 h as previously described (10). Blood was centrifuged at 600 × *g* for 10 min at room temperature and plasma was removed. ACK (ammonium–chloride–potassium) lysing buffer (150 mM NH_4_Cl, 12 mM NaHCO_3_, 1 mM EDTA, pH 7.4) was added to the blood and incubated for 5 min at room temperature and an additional 5 min on ice. Twenty-five milliliters of phosphate-buffered saline (PBS) was added and the cells then centrifuged (500 × *g*, 5 min). This step was repeated three times. After lysis of the red blood cells, basophils were purified to >95% by magnetic negative selection following the manufacturer’s instructions using the Human Basophils Enrichment kit (STEMCELL Technologies, Vancouver, Canada).

### *Ex Vivo* Primary Cell Stimulation

Human blood basophils and mouse splenocytes were cultured in a culture medium (RPMI 1640 with Glutamax and 20 mM HEPES, 1 mM Na-pyruvate, and 1× non-essential amino acids; all from Life Technologies, Carlsbad, CA, USA), 100 μg/ml streptomycin and 100 U/ml penicillin (GE Healthcare, Chicago, IL, USA), and 37.5 μM β-mercaptoethanol (Sigma-Aldrich, St. Louis, MO, USA) supplemented with 20% heat-inactivated fetal calf serum (Life Technologies) at 37°C and 5% CO_2_. For human basophils, 10,000 cells in 200 µl medium per point were used. For mouse splenocytes, 2 million cells in 200 µl medium per point were used. The cells were pretreated or not with 1 µM AMG853 (Tocris, Bio-Techne) over 15 min at 37°C and 5% CO_2_. Then, 1 µM PGD_2_ (Cayman Chemicals, Ann Arbor, MI, USA) was added or not to the cells for 20 h. At the end of the incubation, the cells were harvested and stained as described in the following section.

### Flow Cytometry

Single-cell suspensions from the spleen or peripheral lymph nodes (LNs; pooling inguinal, axillary, and cervical LNs) were prepared as previously described (10). For murine cells, unspecific antibody-binding sites were saturated with a blocking buffer containing 10 µg/ml of anti-CD16/CD32 antibody clone 2.4G2 (BioX Cell, Lebanon, NH, USA) and 100 µg/ml of polyclonal rat IgG, polyclonal mouse IgG, and polyclonal Armenian hamster IgG (Innovative Research Inc., Novi, MI, USA) in fluorescence-activated cell sorting (FACS) buffer (PBS, 1% bovine serum albumin, 1 mM EDTA, and 0.05% sodium azide). Mouse cells were stained in optimized concentrations of fluorophore-conjugated monoclonal antibodies, the list of which is available in **Supplementary Table S1**. Basophils were defined as CD45^lo^CD3^−^CD19^−^CD117^−^CD200R3^+^CD49b^+^FcϵRIα^+^CD123^+^ cells among CD45^+^ viable singlets. Plasmablasts were defined as CD45^+^CD3^−^SSC^lo^CD138^+^CD19^+^I-A/I-E^+^. The ratio of the geometric mean fluorescence intensity (gMFI) of the marker of interest to the isotype control gMFI was normalized to the mean of the values from WT animals treated with vehicle in each experiment and is expressed in arbitrary units (a.u.). Human basophils were stained with the antibodies listed in **Supplementary Table S1**. Human basophils were defined as FcϵRIα^+^CD123^+^CCR3^+^ cells. For all flow cytometry experiments, dead cells were stained in PBS with Ghost 510 viability dye (TONBO Bio., San Diego, CA, USA) and were excluded from the analysis. Before staining, unspecific antibody-binding sites were saturated with a blocking buffer containing 100 µg/ml of polyclonal rat IgG, polyclonal mouse IgG, polyclonal goat IgG, and polyclonal human IgG (Innovative Research Inc.) in FACS buffer. Flow cytometry acquisition was realized using a Becton Dickinson 5-laser LSR II Fortessa X-20 and data analysis using FlowJo vX (Treestar, BD Biosciences, Franklin Lakes, NJ, USA).

### Tissue Analyses and Miscellaneous Assays

Both kidneys were collected. The left kidney was embedded in OCT (CellPath, Powys, UK) and snap-frozen in liquid nitrogen before immunofluorescence analyses. Thereafter, 4-µm acetone-fixed cryosections were blocked in 10% fetal calf serum and stained with fluorescein isothiocyanate (FITC)-conjugated anti-mouse C3 (Cedarlane, Ontario, Canada) or Alexa Fluor^®^ 488-conjugated anti-mouse IgG F(ab)′2 (Jackson ImmunoResearch, West Grove, PA, USA), or their respective isotype controls, before being mounted in Immu-Mount (Thermo Fischer Scientific, Waltham, MA, USA) and analyzed by fluorescent microscopy (Leica DMR; Leica Microsystems, Wetzlar, Germany). The ratio of specific glomerular fluorescence to the tubulointerstitial background was then measured using ImageJ software (NIH, Bethesda, MD, USA), averaging 30 glomeruli per mouse for each sample. Half of the right kidney was homogenized in PBS containing protease inhibitors (Thermo Fischer Scientific) and centrifuged for 10 min at 10,000 × *g* at 4°C. Supernatants were harvested and stored at −80°C until kidney cytokine analyses by ELISA. Mouse interleukin 1β (IL-1β) and IL-4 ELISAs (Duoset; R&D Systems, Minneapolis, MN, USA) were performed as per the manufacturer’s instructions after diluting the kidney extracts to 5 mg/ml of proteins (Pierce BCA Protein Assay, Thermo Fischer Scientific). The other half of the right kidney was fixed in 10% formalin (Sigma-Aldrich), embedded in paraffin, and 5-µm sections were stained by hematoxylin and eosin or Masson’s trichrome and then imaged with a conventional optical microscope (Leica DMD108, Leica Microsystems).

Serum IgE was quantified using a Mouse IgE ELISA Kit (Bethyl Laboratories, Montgomery, TX, USA) with the samples diluted 1:10 or 1:20 and the anti-dsDNA IgG using a homemade method as previously described with the samples diluted 1:50 (10). Absorbance at 450 nm and its correction at 570 nm were assessed by an Infinite 2000 PRO plate reader (Tecan, Männedorf, Switzerland).

### Statistics

**Figure 1:**
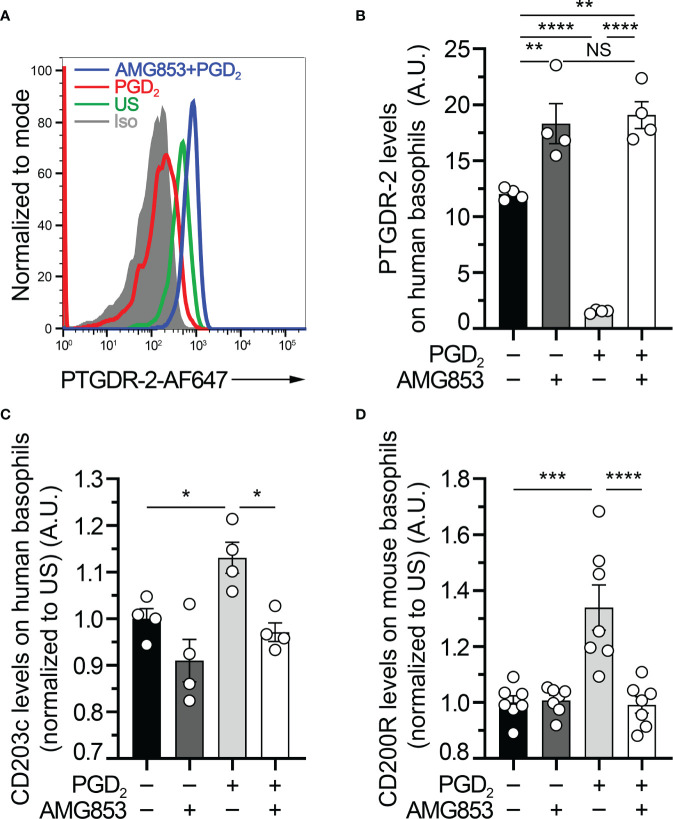
AMG853 blocks *ex vivo* prostaglandin D_2_ (PGD_2_)-induced basophil activation. **(A)** Representative FACS analysis of the expression levels of PTGDR-2 (also CD294 or CRTH2) on purified human blood basophils (as described in *Materials and Methods*) after 20 h of incubation without (−) or with (+) 1 µM PGD_2_ and with or without 1 µM of the bispecific PTGDR-1 and PTGDR-2 antagonist AMG853. *Gray-filled histogram*: isotype control signal; *green line*: unstimulated (US) control; *red line*: PGD_2_-stimulated basophils; *blue line*: AMG853-treated and PGD_2_-stimulated basophils. Human basophils were defined as FcϵRIα^+^CD123^+^CCR3^+^ cells. **(B)** PTGDR-2 levels on purified human basophils treated as indicated in **(A)**. **(C)** CD203c levels on purified human basophils treated as indicated in **(A)** and normalized to the mean of the US conditions. **(D)** CD200R1 (CD200R) levels on mouse basophils from splenocytes from wild-type (WT) mice incubated for 20 h as indicated in **(A)** (as described in *Materials and Methods*) and normalized to the mean of the US conditions. **(B–D)** Results were from at least two independent experiments. Individual values are indicated *inside bars* representing the mean ± SEM. Statistical analysis used one-way ANOVA coupled with Tukey’s multiple comparisons posttest between the indicated groups. ^NS^ *p* > 0.05; *p < 0.05; **p < 0.01; ***p < 0.001; ****p < 0.0001.

We applied Student’s unpaired *t*-tests to compare the differences of one variable between two groups when the distributions were Gaussian and the Mann–Whitney *U* test for non-parametric distributions. When more than two groups were compared (**Figure 1**), one-way ANOVA coupled with Tukey’s multiple comparisons test was used. Paired two-way ANOVA coupled with Holm–Sidak’s posttest was used to analyze the effects of two variables, such as treatment and time, on the same individuals. Individual mice were always represented as a dot, with the mean ± SEM or bars indicating variability from day 0 (D0) to D10 of treatment. Statistical calculations were done using Prism v9 software (GraphPad Software, San Diego, CA, USA).

## Results

### AMG853 Blocks *Ex Vivo* PGD_2_-Induced Basophil Activation

PTGDR-2, also known as CD294 or chemoattractant receptor-homologous molecule expressed on Th2 cells (CRTH2), is internalized following engagement by PGD_2_ (10, 18). Human basophil stimulation by PGD_2_ induced an upregulation of the basophil activation marker CD203c (10, 19). To validate the efficacy of AMG853 in blocking the PGD_2_-induced basophil activation, purified human basophils were pretreated or not with 1 µM of AMG853 and then stimulated with 1 µM PGD_2_ over 20 h. AMG853 pretreatment prevented PGD_2_-induced PTGDR-2 internalization and led to its accumulation on the surface of basophils (**Figures 1A, B**), suggesting that the levels of PTGDR-2 detected in unstimulated conditions were lowered by an autocrine effect of culture-induced PGD_2_ production by basophils, as previously shown (10). PGD_2_ induced an increase in the basophil expression of CD203c, an effect that was completely blocked by AMG853 pretreatment (**Figure 1C**). CD200R (or CD200R1) is a recognized mouse basophil activation marker (20, 21). A similar blocking effect of AMG853 was observed on the PGD_2_-induced CD200R overexpression by mouse spleen basophils (**Figure 1D**).

Together, these results showed that AMG853 could indeed prevent the PGD_2_-induced activation of both human and mouse basophils *ex vivo*.

### AMG853 Treatment Dampens Basophil Accumulation and Activation in SLOs During Lupus-Like Disease

**Figure 2:**
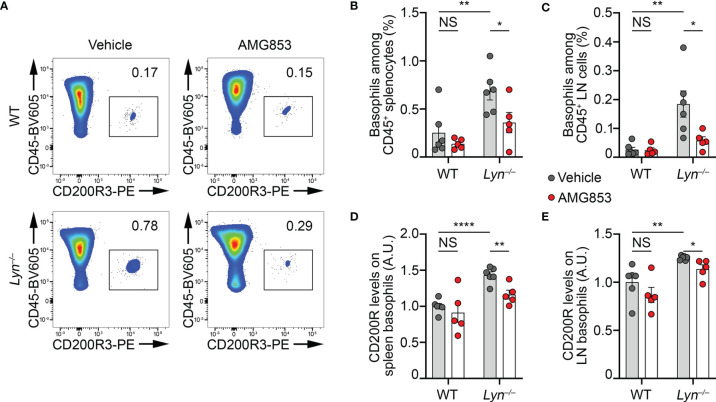
AMG853 dampens the accumulation and activation of basophils in secondary lymphoid organs from *Lyn* ^−/−^ mice. Aged (40–50 weeks) wild-type (WT) and *Lyn* ^−/−^ female mice were treated for 10 days by oral gavage with AMG853 (*n* = 5 per genotype, *red filled circles*) or vehicle (10% EtOH in tap water; *n* = 6 per genotype, *gray filled circles*). Basophil recruitment in the spleen **(A, B)** and peripheral lymph nodes (LNs) **(C)** was assessed by flow cytometry, as exemplified in **(A)** and described in *Materials and methods*. Basophil activation was assessed by measuring the expression levels of CD200R by flow cytometry. The ratio of the geometric mean fluorescence intensity (gMFI) of CD200R signal to the isotype control gMFI was normalized to the mean of the values from WT animals treated with vehicle in each experiment and expressed in arbitrary units (a.u.). Basophil activation was assessed in the spleen **(D)** and LNs **(E)**. Results were from three independent experiments. Individual values are indicated *inside bars* representing the mean ± SEM. Statistical analysis used unpaired Student’s *t-tests* between the indicated groups. ^NS^ *p* > 0.05; *p < 0.05; **p < 0.01; ****p < 0.0001.

*Lyn* ^−/−^ mice have a peripheral basophilia associated with an IgE-, IL-4- and basophil-dependent T helper type 2 (TH2) bias (10, 11). With aging, the basophils of *Lyn* ^−/−^ mice accumulate in SLOs, which support autoreactive humoral immunity, IgE class switching, and the development of a spontaneous lupus-like disease (8, 10, 22, 23). To evaluate the efficacy of AMG853 in reducing the severity of lupus-like disease in sick *Lyn* ^−/−^ mice, 40- to 50-week-old WT and *Lyn* ^−/−^ female mice received treatment by oral gavage with 14 mg kg^−1^ day^−1^ of AMG853 for 10 days. Such treatment led to a dramatic decrease in the detection of basophils in both the spleen and LNs of aged and diseased *Lyn* ^−/−^ mice (**Figures 2A–C** and **Supplementary Figure S1**), confirming the PTGDR-dependent accumulation of basophils in SLOs during the course of the disease in *Lyn* ^−/−^ mice.

The expression of the mouse basophil activation marker CD200R was increased on the surface of basophils in the pristane-induced lupus-like mouse model (9). Similarly, the expression of CD200R was increased on the surface of basophils in the spleen and LNs of aged *Lyn* ^−/−^ mice compared to their WT counterparts, and 10 days of AMG853 treatment decreased its levels on both *Lyn* ^−/−^ spleen and LN basophils (**Figures 2D, E**).

Altogether, these results demonstrated that AMG853 treatment was efficient in dampening basophil activation and accumulation in SLOs in aged and diseased *Lyn* ^−/−^ mice.

### AMG853 Treatment Dampens Plasmablast Accumulation, Autoantibody Titers, and TH2 Environment During Lupus-Like Disease

**Figure 3:**
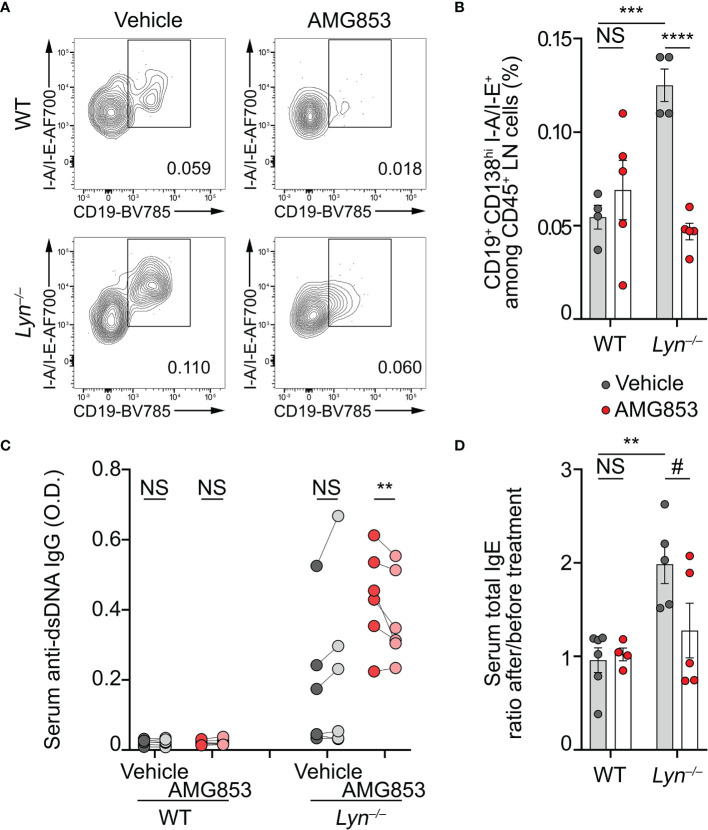
AMG853 dampens plasmablast accumulation, autoantibody titers, and the TH2 environment in *Lyn* ^−/−^ mice. Aged (40–50 weeks) wild-type (WT) and *Lyn* ^−/−^ female mice were treated daily for 10 days by oral gavage with AMG853 (*n* = 5 per genotype, *red filled circles*) or vehicle (10% EtOH in tap water; *n* = 4 per genotype, *gray filled circles*). **(A, B)** Plasmablasts quantified in peripheral lymph nodes (LNs) from the indicated animals by flow cytometry, as exemplified in **(A)** (pre-gated on living CD45^+^CD138^hi^ singlets) and summarized in **(B)**. Plasmablasts were defined as CD45^+^CD138^hi^CD19^+^I-A/I-E^+^ cells among CD45^+^ viable singlets from LN cells. **(C)** Anti-dsDNA IgG autoantibody titers from serum samples harvested before (*dark gray*- and *dark red-filled circles*) and after (*light gray*- and *light red-filled circles*) treatment of each mouse with either vehicle (*gray filled circles*) or AMG853 (*red filled circles*). Individual values for each mouse before and after treatment are represented and linked. *O.D.*, optical density. **(D)** Total IgE titers were determined by ELISA in the serum from the indicated individuals. The ratio between individual values after treatment and values before treatment are represented. Results are from two **(B)** or three **(C, D)** independent experiments. **(B, D)** Individual values are shown *inside bars* representing the mean ± SEM. **(B, D)** Statistical analyses by unpaired Student’s *t*-tests between the indicated groups. **(C)** Statistical analyses done using two-way analysis of variance (ANOVA) followed by Holm–Šídák’s multiple comparisons test. ^NS^ *p* > 0.05; ^#^ *p* = 0.08; **p < 0.001; ***p < 0.001; ****p < 0.0001.

Basophils are known to promote humoral responses through antibody-secreting cell support (8–10, 24, 25). We previously showed that basophils promoted the number and maturation of autoreactive plasmablast in lupus-like mouse models (8–10, 12). Plasmablasts produce autoantibodies of various isotypes, including autoreactive IgG and IgE, which are described as contributing pathogenic factors during SLE (2). As AMG853 dampened the activation and recruitment of basophils to SLOs (**Figures 1**, **2**), we next sought to verify whether AMG853 would also decrease the accumulation of plasmablasts in SLOs and the titers of circulating anti-dsDNA autoantibodies. As anticipated, AMG853 treatment led to a dramatic decrease in the proportions of plasmablast (defined as CD45^+^CD138^hi^CD19^+^I-A/I-E^+^) in the LNs of diseased *Lyn* ^−/−^ mice (**Figures 3A, B**).

As a consequence, while vehicle-treated *Lyn* ^−/−^ mice tended to increase their anti-dsDNA IgG autoantibody levels during the 10 days of experiment, AMG853-treated *Lyn* ^−/−^ mice showed significantly reduced titers of anti-dsDNA IgG autoantibodies over the same period (**Figure 3C**).

Basophils control a constitutive TH2 skewing in *Lyn* ^−/−^ mice in an IgE- and IL-4-dependent manner, which contributes to the development of lupus-like nephritis (8, 11). The serum IgE titers reflected this basophil- and IL-4-dependent TH2 skewing (8). We next measured the total IgE levels as a surrogate marker of the TH2 environment in sera from WT and *Lyn* ^−/−^ mice treated with vehicle or AMG853. During the course of the experiments, vehicle-treated *Lyn* ^−/−^ mice showed a rise in their IgE titers, whereas most of the AMG853-treated *Lyn* ^−/−^ mice had decreased total IgE serum levels, evidencing the induced reduction of the TH2 component of the disease. Importantly, WT mice were not affected by the treatment (**Supplementary Figure S2** and **Figure 3D**).

Altogether, these results gave evidence of the efficacy of targeting PTGDR with the bispecific antagonist AMG853 on reducing both autoantibody-producing cells and autoantibody titers.

### AMG853 Treatment Dampens Immune Complex Deposition, TH2, and Pro-Inflammatory Cytokine Environment in Lupus-Like Nephritis

**Figure 4:**
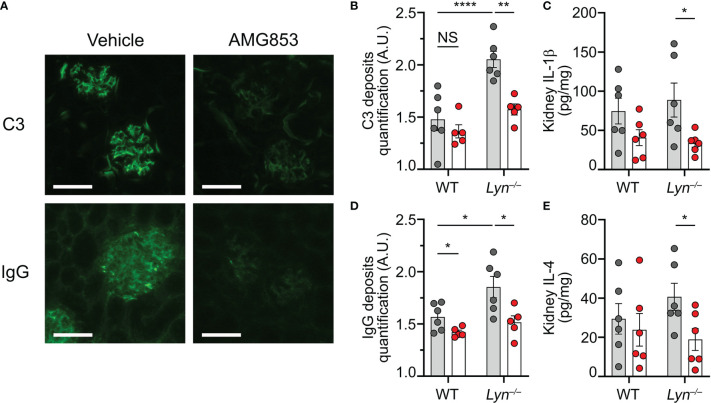
AMG853 dampens lupus-like nephritis in *Lyn* ^−/−^ mice. Aged (40–50 weeks) wild-type (WT) and *Lyn* ^−/−^ mice were treated daily for 10 days by oral gavage with AMG853 (*n* = 5–6 per genotype, *red filled circles*) or vehicle (10% EtOH in tap water; *n* = 6 per genotype, *gray filled circles*). **(A–C)** Cryosections of kidneys (4 µm) analyzed by immunofluorescence for C3 and IgG staining as exemplified for *Lyn* ^−/−^ mice in **(A)** (*scale bar* = 60 µm) and quantified in **(B, C)** as arbitrary units (a.u.) corresponding to the ratio of glomerular measured fluorescence intensity to the interstitial background fluorescence intensity. **(D, E)** Protein extracts from kidneys assessed for total protein and cytokine contents by bicinchoninic acid (BCA) assay and ELISA, respectively, as described in *Materials and methods*. **(D, E)** Content of IL-1β **(D)** or IL-4 **(E)** in kidney extracts expressed in picograms per milligram of renal proteins. **(B–E)** Results from three independent experiments presented as individual values in *bars* representing the mean ± SEM. Statistical analyses used unpaired Student’s *t*-tests between the indicated groups. ^NS^ *p* > 0.05; *p < 0.05; **p < 0.01; ****p < 0.0001.

Beyond the efficacy of AMG853 on the accumulation of basophils and autoantibody-producing cells in SLOs, we next sought to verify whether these effects were associated with a reduction in lupus-like nephritis activity in *Lyn* ^−/−^ mice. As CIC glomerular deposition facilitates inflammation in lupus-like nephritis, we quantified the IgG and complement component C3 deposits in the glomeruli of WT and *Lyn* ^−/−^ mice treated or not with AMG853. AMG853 treatment led to a marked decrease in CIC detection in the glomeruli of treated *Lyn* ^−/−^ mice compared to their vehicle-treated counterparts (**Figures 4A–C**).

We previously showed that basophil depletion in aged *Lyn* ^−/−^ mice led to a dramatic decrease in glomerular CIC deposition and the kidney content of pro-inflammatory cytokines (8, 10). In line with the effects on the accumulation of basophils and the production of autoantibodies, AMG853 treatment significantly reduced the contents of TH2 and pro-inflammatory cytokines (IL-4 and IL-1β) in the kidneys of *Lyn* ^−/−^ mice (**Figures 4D, E**). Of note is that treatment with AMG853 over 10 days was not long enough to ameliorate the glomerular histological lesions observed in aged *Lyn* ^−/−^ mice with established disease (**Supplementary Figure S3**).

These results validated the efficacy of AMG853 treatment in reducing both kidney CIC glomerular deposits and renal inflammation in diseased *Lyn* ^−/−^ mice.

## Discussion

We previously demonstrated the contribution of basophils and the TH2 environment, including IgE and autoreactive IgE, in immune dysregulation leading to the amplification of SLE and lupus nephritis activity. These findings constitute a promising area of new therapeutic strategies for SLE in preclinical and clinical studies (6, 8–10, 12, 26–29). We have recently identified PTGDRs as promising therapeutic targets by assessing, successfully, the efficacy of the combination of two single antagonists, one targeting PTGDR-1 (laropiprant) and the other targeting PTGDR-2 (CAY10471), in ameliorating the autoimmune and renal parameters in lupus-like disease and the importance of the PGD_2_ axis in the pathogenesis of lupus (10). However, AMG853, as a single molecule with a bispecific antagonist activity, would be more easily validated in clinical trials than combined treatments. To develop the translation into clinics of our previous results, we explored the proof of concept that AMG853 was efficient in dampening lupus-like disease in mice.

Here, we provided evidence that AMG853, a bispecific antagonist targeting both PTGDRs, was effective in blocking PGD_2_-induced human and mouse basophil activation *ex vivo* and in controlling basophil recruitment to SLOs, humoral autoimmunity, IgE production, CIC glomerular deposition, and kidney inflammation in aged *Lyn* ^−/−^ mice with established lupus-like nephritis. Then, we provided the proof of concept that this antagonist bispecific for PTGDR1 and PTGDR2 is efficient in dampening the symptoms of lupus-like nephritis in *Lyn* ^−/−^ mice.

Altogether, the increased total IgE titers in the sera of SLE patients, autoreactive IgE, basophil activation and accumulation in SLOs, and the dysregulation of humoral immunity in SLE patients underlined the key role of type 2 immunity in disease activity amplification and increased risks of relapse. As initially developed for atopic diseases, especially with lung involvement, PTGDR antagonists obviously represent a logical approach to control this TH2 side of the disease. If AMG853 failed to show any benefits as an add-on to corticosteroid therapy for patients with moderate to severe asthma, it was well tolerated without any reported serious adverse events over 12 weeks of treatment (16). However, corticosteroid therapy is known to affect the basophil compartment (30), which suggests that any effects of AMG853 on the activation of basophil in the context of asthma may have been missed in this trial. Thus, AMG853 appeared safe as maintenance therapy for lupus nephritis patients at risk of relapse since these patients are in dire need to reduce the morbidity of their long-term immunosuppressive and corticoid treatments. Breaking the basophil-, IgE-, and PGD_2_-dependent amplification loop of SLE might indeed lead to preventing the occurrence of disease flares and also prevent or limit the development of lupus nephritis.

In conclusion, the present study identified AMG853 as a promising candidate to further develop the targeting of PTGDRs in SLE patients. This approach was successfully implemented in *Lyn* ^−/−^ mice in this study, but it needs to be validated in other lupus-prone mouse models such as MRL-*Fas^lpr^* or NZBxNZW F1 mice, which constitute lupus-like mouse models with different pathophysiological origins (31). These validations in other preclinical models might strengthen the concept and allow clinical development of the proposed approach. Another question needs to be addressed. Indeed, here and in previous studies, we analyzed the effects of PTGDR blockade on mice with established lupus-like disease (10). It will be of primary interest to determine in longer-term studies whether PTGDR blockade during the early stage of lupus-like nephritis prevents its development and could then be developed as a preventive therapy for SLE patients as well. These additional preclinical studies might allow developing this approach in clinical studies in the near future.

## Data Availability Statement

The raw data supporting the conclusions of this article will be made available by the authors, without undue reservation.

## Ethics Statement

The studies involving human participants were reviewed and approved by the Comité Régional de Protection des Personnes (CRPP, Paris, France) under the reference ID-RCB 2014-A00809-38. The patients/participants provided written informed consent to participate in this study. The animal study was reviewed and approved by Comité d’éthique Paris Nord no. 121—Ministère de l’enseignement supérieur, de la recherche et de l’innovation under authorization number APAFIS#14115.

## Author Contributions

CP and NC conceived the project, designed and conducted the experiments, and wrote the manuscript. NC directed the project. JT, YL, and CS conducted the experiments. CP and NC had full access to all of the data in the study and take responsibility for the integrity of the data and the accuracy of the data analysis. All authors approved the final version of the article.

## Funding

This work was supported by INSERM Transfert to N.C., the Fondation pour la Recherche Médicale (FRM) (grant no. EQU201903007794 to NC), the French Agence Nationale de la Recherche (ANR) (grant nos. ANR-19-CE17-0029 BALUMET to NC and ANRPIA-10-LABX-0017 INFLAMEX), the Centre National de la Recherche Scientifique (CNRS), by Université de Paris and by the Institut National de la Santé et de la Recherche Médicale (INSERM).

## Conflict of Interest

CP and NC are co-inventors of the patent WO2016128565A1 related to the use of PTGDR-1 and PTGDR-2 antagonists for the prevention or treatment of systemic lupus erythematosus. NC holds another patent related to compositions and methods for treating or preventing lupus (W020120710042).

The remaining authors declare that the research was conducted in the absence of any commercial or financial relationships that could be construed as a potential conflict of interest.

## Publisher’s Note

All claims expressed in this article are solely those of the authors and do not necessarily represent those of their affiliated organizations, or those of the publisher, the editors and the reviewers. Any product that may be evaluated in this article, or claim that may be made by its manufacturer, is not guaranteed or endorsed by the publisher.

## References

[1] Kaul A Gordon C Crow MK Touma Z Urowitz MB van Vollenhoven R. Systemic Lupus Erythematosus. Nat Rev Dis Primers (2016) 2:16039. doi: 10.1038/nrdp.2016.39 27306639

[2] Dema B Charles N. Autoantibodies in SLE: Specificities, Isotypes and Receptors. Antibodies (Basel) (2016) 5(1):2. doi: 10.3390/antib5010002 PMC6698872 31557984

[3] Dema B Charles N. Advances in Mechanisms of Systemic Lupus Erythematosus. Discovery Med (2014) 17(95):247–55. 24882716

[4] Anders HJ Saxena R Zhao MH Parodis I Salmon JE Mohan C. Lupus Nephritis. Nat Rev Dis Primers (2020) 6(1):7. doi: 10.1038/s41572-019-0141-9 31974366

[5] Dorner T Furie R. Novel Paradigms in Systemic Lupus Erythematosus. Lancet (2019) 393(10188):2344–58. doi: 10.1016/S0140-6736(19)30546-X 31180031

[6] Charles N. Autoimmunity, IgE and FcepsilonRI-Bearing Cells. Curr Opin Immunol (2021) 72:43–50. doi: 10.1016/j.coi.2021.03.003 33819742

[7] Charles N Dema B Rivera J. Reply to: Basophils From Humans With Systemic Lupus Erythematosus do Not Express MHC-II. Nat Med (2012) 18:489–90. doi: 10.1038/nm.2664 22481402

[8] Charles N Hardwick D Daugas E Illei GG Rivera J. Basophils and the T Helper 2 Environment can Promote the Development of Lupus Nephritis. Nat Med (2010) 16(6):701–7. doi: 10.1038/nm.2159 PMC2909583 20512127

[9] Dema B Lamri Y Pellefigues C Pacreau E Saidoune F Bidault C. Basophils Contribute to Pristane-Induced Lupus-Like Nephritis Model. Sci Rep (2017) 7(1):7969. doi: 10.1038/s41598-017-08516-7 28801578 PMC5554199

[10] Pellefigues C Dema B Lamri Y Saidoune F Chavarot N Loheac C. Prostaglandin D2 Amplifies Lupus Disease Through Basophil Accumulation in Lymphoid Organs. Nat Commun (2018) 9(1):725. doi: 10.1038/s41467-018-03129-8 29463843 PMC5820278

[11] Charles N Watford WT Ramos HL Hellman L Oettgen HC Gomez G. Lyn Kinase Controls Basophil GATA-3 Transcription Factor Expression and Induction of Th2 Cell Differentiation. Immunity (2009) 30(4):533–43. doi: 10.1016/j.immuni.2009.02.008 PMC2772996 19362019

[12] Dema B Charles N Pellefigues C Ricks TK Suzuki R Jiang C. Immunoglobulin E Plays an Immunoregulatory Role in Lupus. J Exp Med (2014) 211(11):2159–68. doi: 10.1084/jem.20140066 PMC4203948 25267791

[13] Dema B Pellefigues C Hasni S Gault N Jiang C Ricks TK. Autoreactive IgE is Prevalent in Systemic Lupus Erythematosus and is Associated With Increased Disease Activity and Nephritis. PloS One (2014) 9(2):e90424. doi: 10.1371/journal.pone.0090424 24587356 PMC3938730

[14] Labrecque P Roy SJ Frechette L Iorio-Morin C Gallant MA Parent JL. Inverse Agonist and Pharmacochaperone Properties of MK-0524 on the Prostanoid DP1 Receptor. PloS One (2013) 8(6):e65767. doi: 10.1371/journal.pone.0065767 23762421 PMC3677937

[15] Mathiesen JM Christopoulos A Ulven T Royer JF Campillo M Heinemann A. On the Mechanism of Interaction of Potent Surmountable and Insurmountable Antagonists With the Prostaglandin D2 Receptor CRTH2. Mol Pharmacol (2006) 69(4):1441–53. doi: 10.1124/mol.105.017681 16418339

[16] Busse WW Wenzel SE Meltzer EO Kerwin EM Liu MC Zhang N. Safety and Efficacy of the Prostaglandin D2 Receptor Antagonist AMG 853 in Asthmatic Patients. J Allergy Clin Immunol (2013) 131(2):339–45. doi: 10.1016/j.jaci.2012.10.013 23174659

[17] Chan VW Meng F Soriano P DeFranco AL Lowell CA. Characterization of the B Lymphocyte Populations in Lyn-Deficient Mice and the Role of Lyn in Signal Initiation and Down-Regulation. Immunity (1997) 7(1):69–81. doi: 10.1016/S1074-7613(00)80511-7 9252121

[18] Hirai H Tanaka K Takano S Ichimasa M Nakamura M Nagata K. Cutting Edge: Agonistic Effect of Indomethacin on a Prostaglandin D2 Receptor, CRTH2. J Immunol (2002) 168(3):981–5. doi: 10.4049/jimmunol.168.3.981 11801628

[19] Monneret G Boumiza R Gravel S Cossette C Bienvenu J Rokach J. Effects of Prostaglandin D(2) and 5-Lipoxygenase Products on the Expression of CD203c and CD11b by Basophils. J Pharmacol Exp Ther (2005) 312(2):627–34. doi: 10.1124/jpet.104.074823 15388786

[20] Bakocevic N Claser C Yoshikawa S Jones LA Chew S Goh CC. CD41 is a Reliable Identification and Activation Marker for Murine Basophils in the Steady State and During Helminth and Malarial Infections. Eur J Immunol (2014) 44(6):1823–34. doi: 10.1002/eji.201344254 24610714

[21] Torrero MN Larson D Hubner MP Mitre E. CD200R Surface Expression as a Marker of Murine Basophil Activation. Clin Exp Allergy (2009) 39(3):361–9. doi: 10.1111/j.1365-2222.2008.03154.x PMC2743132 19134017

[22] Hibbs ML Tarlinton DM Armes J Grail D Hodgson G Maglitto R. Multiple Defects in the Immune System of Lyn-Deficient Mice, Culminating in Autoimmune Disease. Cell (1995) 83(2):301–11. doi: 10.1016/0092-8674(95)90171-X 7585947

[23] Nishizumi H Taniuchi I Yamanashi Y Kitamura D Ilic D Mori S. Impaired Proliferation of Peripheral B Cells and Indication of Autoimmune Disease in Lyn-Deficient Mice. Immunity (1995) 3(5):549–60. doi: 10.1016/1074-7613(95)90126-4 7584145

[24] Denzel A Maus UA Rodriguez Gomez M Moll C Niedermeier M Winter C. Basophils Enhance Immunological Memory Responses. Nat Immunol (2008) 9(7):733–42. doi: 10.1038/ni.1621 18516038

[25] Rodriguez Gomez M Talke Y Goebel N Hermann F Reich B Mack M. Basophils Support the Survival of Plasma Cells in Mice. J Immunol (2010) 185(12):7180–5. doi: 10.4049/jimmunol.1002319 21068399

[26] Charles N Chemouny JM Daugas E. Basophil Involvement in Lupus Nephritis: A Basis for Innovation in Daily Care. Nephrol Dial Transplant (2019) 34(5):750–6. doi: 10.1093/ndt/gfy245 31009949

[27] Pellefigues C Charles N. The Deleterious Role of Basophils in Systemic Lupus Erythematosus. Curr Opin Immunol (2013) 25(6):704–11. doi: 10.1016/j.coi.2013.10.003 PMC3876474 24209595

[28] Halfon M Bachelet D Hanouna G Dema B Pellefigues C Manchon P. CD62L on Blood Basophils: A First Pre-Treatment Predictor of Remission in Severe Lupus Nephritis. Nephrol Dial Transplant (2021) 36(12):2256–62. doi: 10.1093/ndt/gfaa263 33316058

[29] Hasni S Gupta S Davis M Poncio E Temesgen-Oyelakin Y Joyal E. Safety and Tolerability of Omalizumab: A Randomized Clinical Trial of Humanized Anti-IgE Monoclonal Antibody in Systemic Lupus Erythematosus. Arthritis Rheumatol (2019) 71(7):1135–40. doi: 10.1002/art.40828 PMC6594871 30597768

[30] Zen M Canova M Campana C Bettio S Nalotto L Rampudda M. The Kaleidoscope of Glucorticoid Effects on Immune System. Autoimmun Rev (2011) 10(6):305–10. doi: 10.1016/j.autrev.2010.11.009 21224015

[31] Zhuang H Szeto C Han S Yang L Reeves WH. Animal Models of Interferon Signature Positive Lupus. Front Immunol (2015) 6:291. doi: 10.3389/fimmu.2015.00291 26097482 PMC4456949

