# Supplementary material for: AMG853, A Bispecific Prostaglandin D2 Receptor 1 and 2 Antagonist, Dampens Basophil Activation and Related Lupus-Like Nephritis Activity in Lyn-Deficient Mice
Source: Front Immunol. 2022 Apr 4;13:824686. doi: 10.3389/fimmu.2022.824686 (PMC9014266; doi:10.3389/fimmu.2022.824686)
Supplement: Supplementary file 1 [file DataSheet_1.docx]

**AMG853, a bispecific Prostaglandin D2 receptors -1 and -2 antagonist, dampens basophil activation and related lupus-like nephritis activity in Lyn deficient mice**

**SUPPLEMENTARY MATERIAL**

**Authors**: Christophe Pellefigues Ph.D.^1,2^, John Tchen M.Sc.^1,2^, Chaimae Saji^1,2^, Yasmine Lamri Ph.D.^1,2^ and Nicolas Charles Ph.D.^1,2^*.

^1^ Université de Paris, Centre de Recherche sur l’Inflammation, INSERM UMR1149, CNRS ERL8252, Faculté de Médecine site Bichat, Paris, France.

^2^ Université de Paris, Laboratoire d’Excellence INFLAMEX, Paris, France.

***Correspondence to**:

Nicolas Charles, PhD

Centre de Recherche sur l’Inflammation, INSERM UMR1149, CNRS ERL8252,

Université de Paris, Faculté de Médecine site Bichat,

16 rue Henri Huchard, 75018 Paris, France.

Phone: +33 157277306

E-mail: [nicolas.charles@inserm.fr](mailto:nicolas.charles@inserm.fr) **SUPPLEMENTARY FIGURE S1**


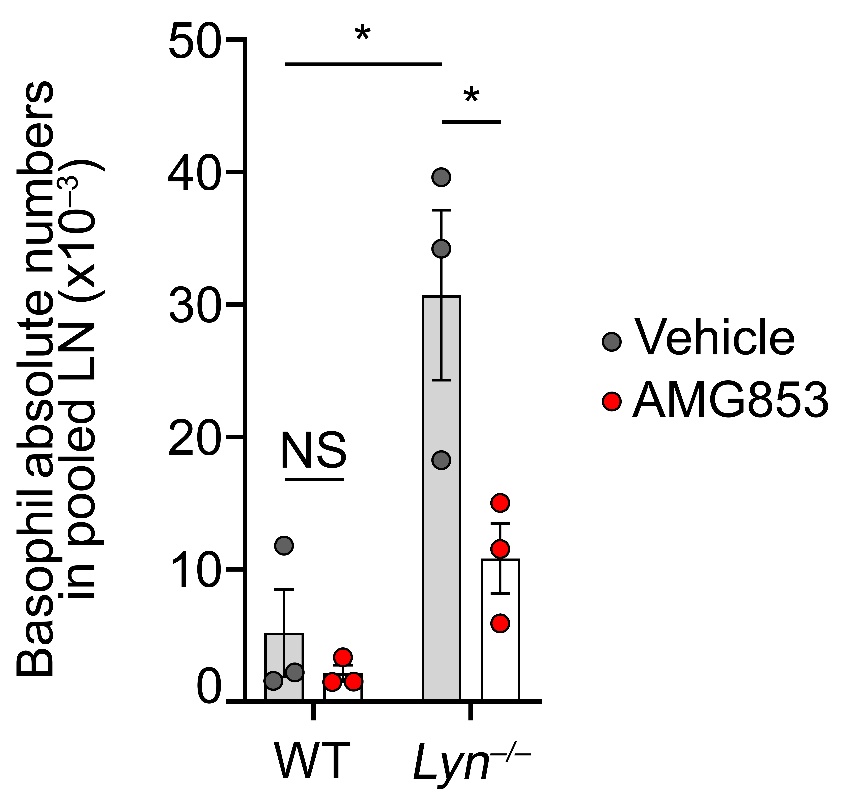


**SUPPLEMENTARY FIGURE S1: AMG853 dampens basophil numbers accumulated in *Lyn^–/–^* mice lymph nodes.**

Aged (40-50 weeks) wild type (WT) and *Lyn^–/–^* female mice were treated for 10 days by oral gavage with AMG853 (n=3 per genotype, red filled circles) or vehicle (10% EtOH in tap water; n=3 per genotype, grey filled circles). Basophil recruitment in pooled peripheral lymph nodes (LN) (cervical, axillary and inguinal lymph nodes (LN)) was assessed by flow cytometry as in **Figure 2**. Total number of cells was assessed with a hemacytometer and proportions of basophils (see Figure 2) were used to determine absolute number of basophils per pool of LN. Results are from two independent experiments. Individual values are indicated inside bars representing the mean ± s.e.m.; Statistical analysis was done by unpaired Student *t-tests* between the indicated groups. NS: p>0.05; *: p<0.05.

**SUPPLEMENTARY FIGURE S2**

**
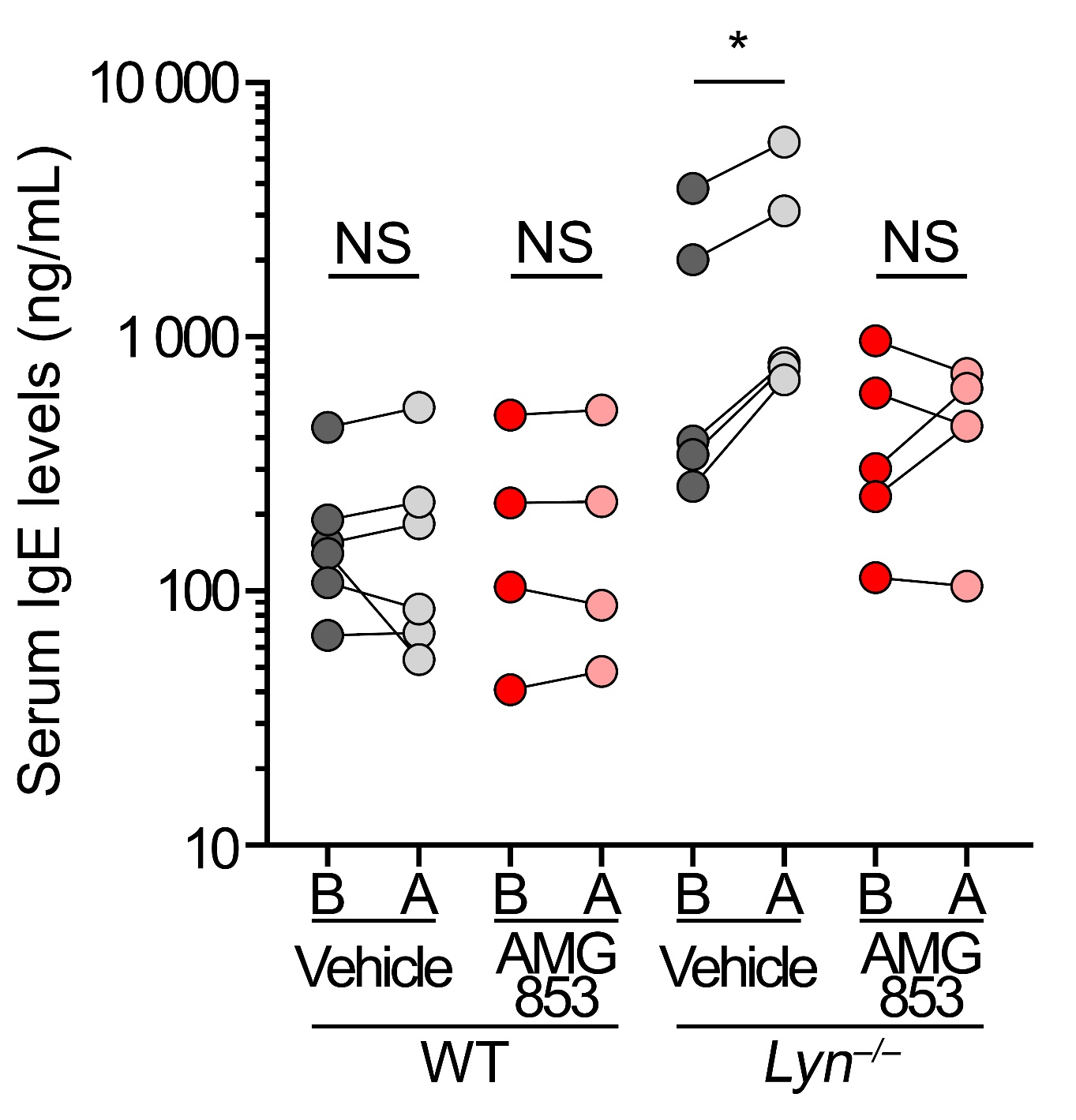
**

**SUPPLEMENTARY FIGURE S2: Serum IgE titers evolution over the treatment period (related to Figure 3D)**

Serum IgE titers were assessed by ELISA in the indicated groups of mice before (B) and after (A) treatment. These values were used to generate the ratios presented in **Figure 3D**. Statistical analysis was done by paired Student *t-tests* between the indicated groups. NS: p>0.05; *: p<0.05.

**SUPPLEMENTARY FIGURE S3**


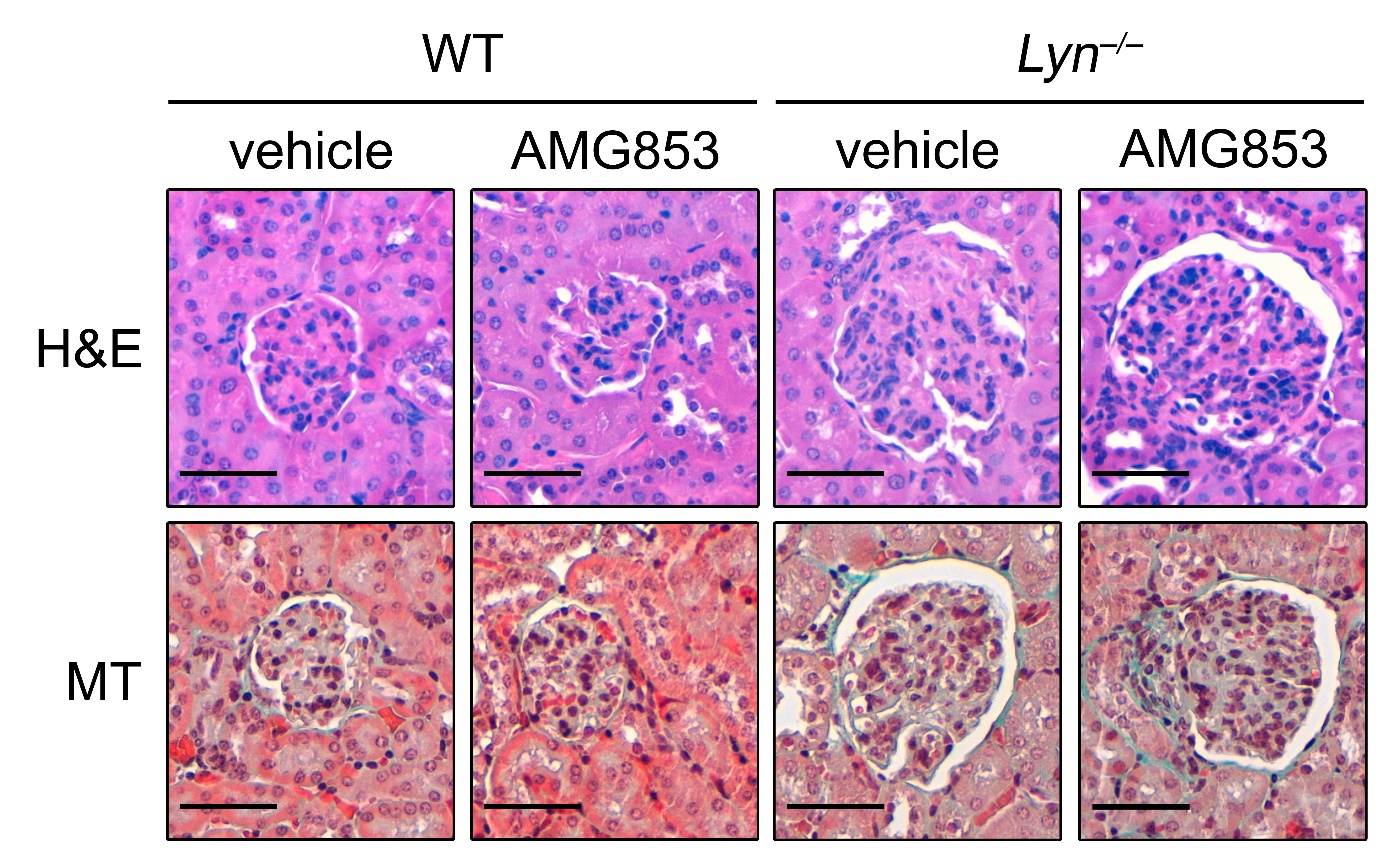


**SUPPLEMENTARY FIGURE S3: Representative glomerular histology of WT and *Lyn^–/–^* kidneys in vehicle or AMG853-treated mice over 10days.**

Formalin-fixed and paraffin-embedded kidney slides from aged wild type (WT) and *Lyn^–/–^* female mice treated for 10 days with vehicle or AMG853 were stained with hematoxylin and eosin (H&E) or Masson’s trichrome (MT). A representative glomerulus from each genotype/treatment is shown as indicated. No difference induced by the 10 days-long treatment was observed on kidney lesions in *Lyn^–/–^* mice Scale bar = 50 µm.

**Supplementary Table S1: Antibodies used for flow cytometry**

| **Fluorophore** | **Mouse Target** | **Clone** | **Isotype** | **Company** |
| --- | --- | --- | --- | --- |
| Alexa Fluor® 647 | FcεRIα | MAR-1 | AH IgG | BioLegend |
| Alexa Fluor® 700 | IA-IE | M5/114 | Rat IgG2b, κ | BioLegend |
| APC | CD138 | 281-2 | Rat IgG2a, κ | BioLegend |
| APC-Cy7 | CD19 | 6D5 | Rat IgG2a, κ | BioLegend |
| APC-Cy7 | CD117 | 2B8 | Rat IgG2b, κ | BioLegend |
| APC-Cy7 | CD3ε | 145-2C11 | AH IgG | BioLegend |
| BV605 | CD45 | 30-F11 | Rat IgG2b, κ | BioLegend |
| BV785 | CD19 | 6D5 | Rat IgG2a, κ | BioLegend |
| FITC | CD123 | 5B11 | Rat IgG2a, κ | eBioscience |
| Pacific Blue^TM^ | CD49b | DX5 | Rat IgM, κ | BioLegend |
| PE | CD200R3 | Ba13 | Rat IgG2a, κ | BioLegend |
| PE-Dazzle^TM^ 594 | CXCR4 | L276F12 | Rat IgG2b, κ | BioLegend |
| PerCP-eFluor^TM^ 710 | CD200R | OX110 | Rat IgG2a, κ | eBioscience |
|  |  |  |  |  |
| **Fluorophore** | **Human Target** | **Clone** | **Isotype** | **Company** |
| Alexa Fluor® 647 | CD294 | BM16 | Rat IgG2a, κ | BioLegend |
| BV421 | CD203c | NP4D6 | Mo IgG1, κ | BioLegend |
| BV605 | CCR3 | 5E8 | Mo IgG2b, κ | BioLegend |
| PE-Dazzle^TM^ 594 | CD123 | 6H6 | Mo IgG1, κ | BioLegend |
| PE-Cy7 | FcεRIα | AER-37 | Mo IgG2b, κ | BioLegend |
|  |  |  |  |  |
| **Fluorophore** | | **Clone** | **Isotype Control** | **Company** |
| Alexa Fluor® 647 | | HTK888 | AH IgG | BioLegend |
| Alexa Fluor® 700 | | RTK4530 | Rat IgG2b, κ | BioLegend |
| APC | | RTK2758 | Rat IgG2a, κ | BioLegend |
| APC-Cy7 | | RTK2758 | Rat IgG2a, κ | BioLegend |
| APC-Cy7 | | RTK4530 | Rat IgG2b, κ | BioLegend |
| APC-Cy7 | | HTK888 | AH IgG | BioLegend |
| BV605 | | RTK4530 | Rat IgG2b, κ | BioLegend |
| BV785 | | RTK2758 | Rat IgG2a, κ | BioLegend |
| FITC | | RTK2758 | Rat IgG2a, κ | BioLegend |
| Pacific Blue^TM^ | | RTK2118 | Rat IgM, κ | BioLegend |
| PE | | RTK2758 | Rat IgG2a, κ | BioLegend |
| PE-Dazzle^TM^ 594 | | RTK4530 | Rat IgG2b, κ | BioLegend |
| PerCP-eF710 | | eBR2a | Rat IgG2a, κ | eBioscience |
| Alexa Fluor® 647 | | RTK2758 | Rat IgG2a, κ | BioLegend |
| BV421 | | MOPC-21 | Mo IgG1, κ | BioLegend |
| BV605 | | MPC-11 | Mo IgG2b, κ | BioLegend |
| PE-Dazzle^TM^ 594 | | MOPC-21 | Mo IgG1, κ | BioLegend |
| PE-Cy7 | | MPC-11 | Mo IgG2b, κ | BioLegend |

Abbreviations used: AH: Armenian Hamster, APC: Allophycocyanin, BV: Brilliant Violet^TM^, Cy: Cyanin, CD: cluster of differentiation, FITC: Fluorescein Isothiocyanate, Mo: Mouse, PE: Phycoerythrin, PerCP: Peridinin Chlorophyll Protein Complex.
